# *In Vivo* Evaluation of TNF-Alpha in the Lungs of Patients Affected by Sarcoidosis

**DOI:** 10.1155/2015/401341

**Published:** 2015-03-19

**Authors:** Filippo Galli, Tiziana Lanzolla, Vittorio Pietrangeli, Gaurav Malviya, Alberto Ricci, Pierdonato Bruno, Paola Ragni, Francesco Scopinaro, Salvatore Mariotta, Alberto Signore

**Affiliations:** ^1^Nuclear Medicine Unit, Department of Medical-Surgical Sciences and Translational Medicine, Faculty of Medicine and Psychology, Ospedale S. Andrea, “Sapienza” University of Rome, Via di Grottarossa 1035, 00189 Roma, Italy; ^2^Pneumology Unit, Department of Clinical and Molecular Medicine, Faculty of Medicine and Psychology, Ospedale S. Andrea, “Sapienza” University of Rome, Via di Grottarossa 1035, 00189 Roma, Italy

## Abstract

*Introduction*. Sarcoidosis is a multisystemic granulomatous disorder characterized by multiple noncaseating granulomas involving intrathoracic lymph nodes and lung parenchyma. Recently, the use of anti-tumor necrosis factor alpha (anti-TNF*α*) agents has been introduced for therapy of chronic and refractory sarcoidosis with controversial results. Infliximab (Remicade) is a chimeric monoclonal antibody (mAb) that recognizes and binds TNF*α*, neutralizing its biological effects. In the present study, _ _^99m^Tc labelled infliximab was used to study the expression of TNF*α* in sarcoid lesions and to evaluate its role as a predictive marker in response to therapy with Remicade. *Material and Methods*. A total of 10 patients with newly diagnosed sarcoidosis were enrolled together with 10 control patients affected by rheumatoid arthritis. All patients were studied by planar imaging of the chest with _ _^99m^Tc-infliximab at 6 h and 24 h and total body [^18^F]-FDG PET/CT. Regions of interest were drawn over the lungs and the right arm and target-to-background ratios were analysed for _ _^99m^Tc-infliximab. SUV_mean_ and SUV_max_ were calculated over lungs for FDG. *Results and Discussion*. Image analysis showed low correlation between *T/B* ratios and BAL results in patients despite positivity at [^18^F]-FDG PET. *Conclusion*. In conclusion, patients with newly diagnosed pulmonary sarcoidosis, with FDG-PET and BAL positivity, showed a negative _ _^99m^Tc-infliximab scintigraphy.

## 1. Introduction

Sarcoidosis is a multisystemic granulomatous disorder characterized by multiple noncaseating granulomas involving intrathoracic lymph nodes and lung parenchyma. Inflammatory lesions can occur also in other organs like eyes and skin and, although less frequently, also in liver, spleen, extrathoracic lymph nodes, salivary glands, heart, nervous system, bones, and muscles. Even if its aetiology is still unknown, the role of cell-mediated immunity in the formation and in the maintenance of typical granulomas has been clarified [1, 2]. For this reason immunosuppressive therapy remains the gold standard for treatment and in particular corticosteroid are used as a first line therapy [3]. However, serious side effects of steroid therapy and the loss of long-term efficacy of this treatment have led researchers to use new drugs. Recently, the use of anti-tumor necrosis factor alpha (anti-TNF*α*) agents has been introduced for therapy of chronic and refractory sarcoidosis [4–6]. TNF*α* is an important cytokine released by alveolar activated macrophages, implicated in the development of granulomas. Infliximab (Remicade) is a chimeric monoclonal antibody (mAb) that recognizes and binds TNF*α*, neutralizing its biological effects [7]. However, the effectiveness of such therapy is still uncertain and under investigation [8]. It is indeed not well known if TNF*α* is sufficiently present in sarcoid lesions to play a relevant biological role despite the fact that its presence in BAL has been shown to correlate with the severity of alveolitis [9]. Nevertheless, which patient and which lesion have high levels of TNF*α* and may respond to anti-TNF*α* therapy is difficult to ascertain. In this view, the assessment of TNF*α* in sarcoid lesions by a noninvasive technique could be important to strengthen the hypothesis behind the use of anti-TNF*α* drugs and to select patients that could respond to therapy. Currently there are no specific diagnostic tools to directly evaluate the presence of anti-TNF*α* in sarcoid lesions but several methods have been reported for measuring disease activity. Chest X-ray and pulmonary function tests (spirometry), the measurement of serum angiotensin-converting-enzyme (ACE) levels and ^67^Ga-citrate scintigraphy, and bronchoalveolar lavage (BAL) with evaluation of CD4^+^ and CD8^+^ lymphocytes have all been and still are used as surrogate markers of diseases activity [10, 11]. In particular, [^18^F]-Fluoro-2-deoxy-D-glucose PET/CT ([^18^F]-FDG PET/CT) has been shown to be of high clinical value for evaluation of disease activity and extent and for therapy follow-up [12–15].

In the present study, we have used ^99m^Technetium (^99m^Tc) labelled infliximab in patients with newly diagnosed sarcoidosis for noninvasive* in vivo* scintigraphic evaluation of the presence of TNF*α* in pulmonary and lymph nodal sarcoid lesions. Patients were also studied by [^18^F]-FDG PET/CT and BAL with lymphocyte phenotyping for complete evaluation of disease activity.

## 2. Patients and Methods

### 2.1. Patients and Diagnosis

Study design included 20 patients with newly diagnosed sarcoidosis at stages II-III to be prospectively recruited for [^18^F]-FDG-PET/CT and ^99m^Tc-infliximab scintigraphy and 10 control subjects (patients without sarcoidosis but affected by rheumatoid arthritis (RA) to evaluate disease activity in joints). After enrolling all controls (7 females and 3 males, mean age 54 ± 10 years) and 10 sarcoidosis patients (8 females and 2 males, mean age 55 ± 8 years) we performed an interim analysis and decided to stop recruitment, based on results. Sarcoidosis patients were symptomatic and presented respiratory symptoms of the disease, without involvement of specific organs but lungs, thoracic and extrathoracic lymph nodes. They were also subjected to a standard assessment that included history and physical examination, with particular attention to respiratory disorders, blood test with peripheral blood counts and lymphocytes count ratio, and X-ray examination of the chest, including X-ray and high-resolution CT, bronchoscopy with bronchoalveolar lavage and bronchial biopsy, and analysis of BAL with lymphocytes immune-phenotyping (2 patients refused to perform the BAL). The diagnosis of sarcoidosis was performed using histological demonstration of the presence of the typical noncaseating granulomas; other diseases such as Wegener's granulomatosis, tuberculosis, aspergillosis, and neoplastic diseases were excluded for each patient. None of enrolled patients had previously been treated with corticosteroid therapy or immunosuppressive drugs. The study was approved by the local medical ethical committee and each patient expressed written informed consensus.

### 2.2. [^18^F]-FDG PET/CT

Within 2 months from clinical diagnosis of sarcoidosis, a [^18^F]-FDG PET/CT was performed after fasting for at least 6 h before the intravenous injection of [^18^F]-FDG and with a serum glucose level lower than 160 mg/dL. Diazepam (5 mg) was administered to reduce muscle activity and activation of the brown fat. The activity of [^18^F]-FDG to be administered was calculated for each patient according to the following formula [(weight in Kg/10 × 37 MBq) + 1]. The PET scan was performed with hybrid PET/CT Gemini (Philips, NL). Imaging acquisition started 60 minutes after the radiopharmaceutical injection from the upper thigh to the head, with a preliminary low-dose unenhanced CT scan (16 slice, 100 mAs) followed by PET imaging (2.5 min per bed position, 3D mode, matrix). Images were reconstructed with CT data by common iterative algorithm (OSEM, ordered subset expectation maximization, 2 iterations, 28 subsets) to obtain attenuation corrected images and anatomical mapping on functional images. [^18^F]-FDG PET/CT images were visually analysed and disease activity was assessed separately in the mediastinum, hilum, lung parenchyma, extrapulmonary lymph nodes, even with obvious evaluation of liver, spleen, bone marrow, bones, and joints, in order to highlight a possible involvement of these organs. Each site was scored either positive or negative (positive = [^18^F]-FDG uptake higher than background; negative = [^18^F]-FDG uptake lower or equal to background). The semiquantitative analysis was based on the analysis of standardized uptake value (SUV) evaluated as SUV_max⁡_ and SUV_mean_, obtained by drawing regions of interest (ROIs) on transaxial sections of lung parenchyma at the level of the 3rd, 5th, and 7th thoracic vertebral body. The SUV values obtained were then compared with those obtained in the control population.

### 2.3. ^99m^Tc-Infliximab Scintigraphy

The mAb infliximab was radiolabelled as previously described [16]. Briefly, 200 *μ*L of ^99m^TcO_4_^−^ (666 MBq) was added to 200 *μ*L of reduced mAb (200 mg/mL) followed by 7 *μ*L of methylene diphosphonate (MDP). After 10 minutes of incubation at room temperature, quality controls were performed by instant thin layer chromatography (ITLC). Silica gel strips and 0.9% NaCl solution were used, respectively, as stationary and mobile phase for labelling efficiency determination. Albumin precoated silica gel strips and H_2_O :  EtOH : NH_3_ (5 : 3 : 1) solution were used for colloids evaluation.

Within 1 week from the PET/CT scan, all patients performed a scintigraphic study with radiolabelled anti-TNF*α* after intravenous injection of 370 MBq of ^99m^Tc-infliximab. Whole body images and planar static images of chest were acquired at 6 h and 24 h after injection with a large field of view, two head, gamma camera (Sky Light, Philips, NL) equipped with low-energy high-resolution collimators and 20% energy windows centred at 140 KeV. Whole body images (matrix 512 × 1024) were acquired at a speed of 10 cm/min at 6 h and 5 cm/min at 24 h taking into account the decay of the radionuclide. Anterior and posterior thorax images (matrix 256 × 256) were acquired for 300 seconds at 6 h and 600 seconds at 24 h.

The results of scintigraphic studies were qualitatively analysed to identify any labelled mAb uptake and were visually compared with the pathological findings on PET images. A semiquantitative analysis was performed drawing ROIs over each lung parenchyma (ROI_lung_) including hilum, but excluding heart and spine, and a ROI over the upper right arm (ROI_arm_) as background, excluding joints. The counts were normalized by area and the target-to-background (*T/B*) ratio (ROI_lung_/ROI_arm_) was calculated at 6 h and 24 h for each lung in anterior and in posterior views. The average value of anterior and posterior values was considered for each patient. Results were compared with control subjects that underwent ^99m^Tc-infliximab for RA and therefore considered “pulmonary negative.”

### 2.4. Statistical Analysis

For each patient the average values of SUV_max⁡_ and SUV_mean_, obtained in lung parenchyma, were calculated in both sarcoidosis patients and controls, obtaining for each group the mean value ± SD. Similarly, the mean values ± SD of* T/B* ratios in sarcoidosis patients and control group were calculated at each time point (6 h and 24 h). In sarcoidosis population correlations were made between mean SUV values and mean* T/B* ratio, mean SUV values and lymphocytes immune-phenotyping on BAL, and mean* T/B* ratio and lymphocytes immune-phenotyping on BAL, in order to assess the diagnostic accuracy of each methodology to evaluate disease activity. Student's *t*-test was applied to assess the significance of relationship between the* T/B* ratio of sarcoidosis group and* T/B* ratio of control subjects.

## 3. Results

### 3.1. [^18^F]-FDG PET/TC

**Figure 1:**
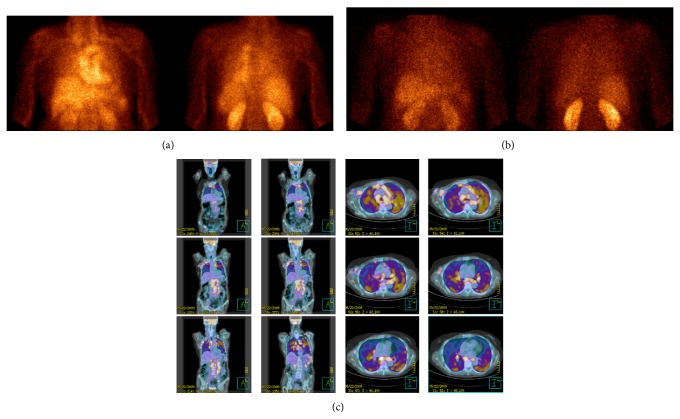
^99m^Tc-Infliximab scintigraphy of a sarcoidosis patient (patient 3/F) acquired at 6 h (anterior and posterior views (a)) and at 24 h (anterior and posterior views (b)) showing a moderate and diffuse uptake in the lung parenchyma. [^18^F]-FDG PET/CT images of the same patient showing a focal/hyleal uptake (coronal and transaxial sections (c)).

The qualitative analysis performed on PET/CT studies in lungs showed complete agreement with the staging previously made by pulmonologists according to radiological and biochemical findings (Figure 1). Furthermore, in 3/10 patients PET showed other extrapulmonary sites of disease, with involvement of axillary and abdominal-pelvic lymph nodes, not previously known, demonstrating its high sensitivity. The mean values ± SD of SUV_max⁡_ obtained in patients with sarcoidosis were 4.43 ± 3.20 whereas in control subjects mean values ± SD of SUV_max⁡_ were 1.18 ± 0.20 (*P* < 0.001). These data are an expression of inflammation involvement of lung parenchyma in patients with sarcoidosis. The values of SUV_max⁡_ and SUV_mean_ correlated perfectly; therefore for subsequent analysis only SUV_max⁡_ will be reported even if also SUV_mean_ has always been considered. Despite the higher values of SUV_max⁡_ in patients with sarcoidosis with respect to controls, it was not possible to find a cut-off value that could allow the correlation of the extent of uptake in the lung parenchyma with the degree of alveolitis. It was not also possible to obtain a significant correlation between lymphocyte immune-phenotyping results of BAL (with particular reference to CD4^+^/CD8^+^ ratio) and the SUV_max⁡_ values of patients with sarcoidosis. No correlation was also observed between SUV_max⁡_ or SUV_mean_ values and the CD4^+^/CD8^+^ ratio in blood.

### 3.2. ^99m^Tc-Infliximab Scintigraphy

**Figure 2:**
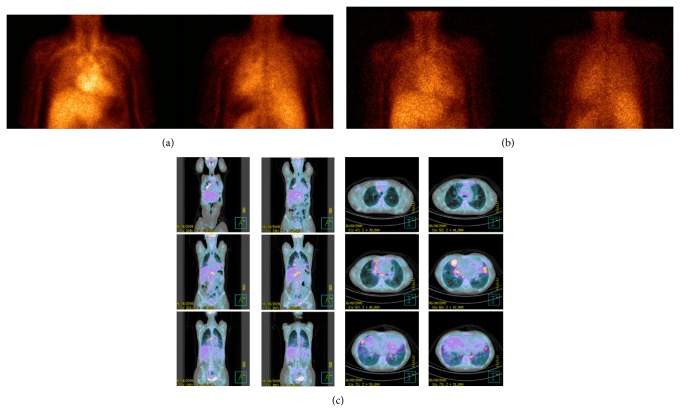
^99m^Tc-Infliximab scintigraphy of a sarcoidosis patient (patient 4/F) acquired at 6 h (anterior and posterior views (a)) and at 24 h (anterior and posterior views (b)) showing a moderate and diffuse uptake in the lung parenchima. [^18^F]-FDG PET/CT images of the same patient showing a focal/hyleal uptake (coronal and transaxial sections (c)).

The qualitative analysis of scintigraphy with ^99m^Tc-infliximab showed no pathological focal accumulation of the labeled antibody (Table 1). Both images at 6 h, characterized by high vascular activity, and images at 24 h did not show the same pathological uptakes highlighted in the preliminary PET study (Figures 1 and 2). The pulmonary distribution of the two radiopharmaceuticals was different, being predominantly diffused in the case of ^99m^Tc-infliximab and rather focal with ileal involvement in the PET scans.

**Figure 3:**
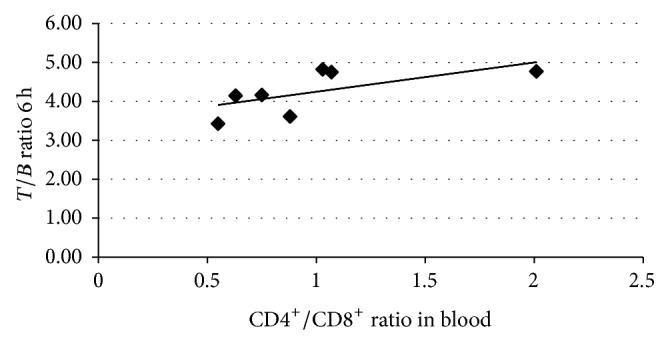
Correlation between lung uptake of ^99m^Tc-infliximab at 6 h (*T/B* ratio) and CD4^+^/CD8^+^ ratio in peripheral blood lymphocytes. Correlation coefficient is *r* = 0.2357 and *P* = 0.05.

**Figure 4:**
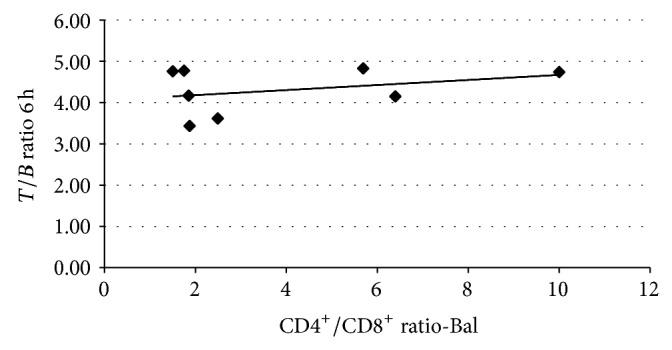
Correlation between lung uptake of ^99m^Tc-infliximab at 6 h (*T/B* ratio) and CD4^+^/CD8^+^ ratio in lymphocytes from BAL. Correlation coefficient is *r* = 0.6487 and *P* = n.s.

Comparing* T/B* ratios on ^99m^Tc-infliximab scintigraphy at 6 h and 24 h, with SUV_max⁡_ values of pulmonary uptake of FDG, no significant correlation was observed between these parameters. In only three patients a detectable diffuse bilateral lung uptake of anti-TNF*α* at 6 h and 24 h on scintigraphic images was present, which could indicate increased levels of TNF*α* in lung parenchyma of these patients. When we compared the mean values of lung uptake of labelled anti-TNF*α* mAb in sarcoidosis patients and control subjects, we found significant differences at 6 h (4.28 ± 0.57 versus 3.2 ± 0.74; patients versus controls; *P* = 0.002) but not at 24 h (3.15 ± 0.45 versus 2.7 ± 0.65; patients versus controls; *P* = 0.057). A moderate correlation was found between CD4^+^/CD8^+^ ratio peripheral blood lymphocytes and the value of* T/B* ratio at 6 h (Figure 3) but no correlation was found between average value of* T/B* ratio at 6 h or 24 h and, respectively, CD4^+^/CD8^+^ ratio and cellularity of BAL (Figure 4).

## 4. Discussion

In the last decade, systemic autoimmune disease therapy has been revolutionized by the availability of biological drugs or monoclonal antibodies, directed against a specific target implicated in the pathogenesis of disease. In patients with active sarcoidosis, the release of TNF*α* by activated alveolar macrophages has been widely documented in a lot of previous studies [17–21]. Infliximab (Remicade) is a chimeric monoclonal antibody of the type IgG1*κ*, with a variable region derived from murine antihuman TNF*α* and a constant sequence of human-derived IgG1. It was one of the first biological drugs to be used for the treatment of patients with sarcoidosis refractory to conventional therapy [5, 6, 22–24]. In particular, the multicenter phase II study of Baughman et al. [25] showed that in patients with sarcoidosis, symptomatic, refractory to corticosteroid therapy, treated with infliximab, there was a significant improvement of the forced vital capacity (FVC) without significant side effects related to the use of the drug. However, Panselinas et al. [26] showed, in a retrospective study, that in the majority of patients treated with infliximab, there was a recurrence of the disease about 3 months after discontinuation of the drug. Since biological therapies, such as infliximab, are extremely expensive, it would be desirable to be able to accurately select patients who really might benefit from this type of therapies. To date, however, there are no diagnostic markers for therapy decision making. In order to answer this question, we undertook a study to assess whether scintigraphy with ^99m^Tc-infliximab, showing directly the presence of TNF*α* in the lesions, may represent a marker for predicting the efficacy of biological therapy with anti-TNF*α* and then select the patients suitable for this type of treatment [27, 28]. Infliximab was labelled with high labelling efficiency, high specific activity, and stability. Preliminary studies* in vitro* in animals and humans have shown its usefulness in the evaluation of patients with Crohn's disease and rheumatoid arthritis [29–31]. [^18^F]-FDG PET has proven to be a very sensitive method in the evaluation of disease activity. Many studies have shown its higher sensitivity compared to ^67^Gallium-citrate scintigraphy in the evaluation of disease and follow-up of therapy [14, 32–38]. However, [^18^F]-FDG PET lacks specificity and cannot be used for the selection of patients to be treated with anti-TNF*α*. Its main role remains the diagnostic confirmation of disease, evaluation of the extension of the sites of disease, and the follow-up. It is therefore right to compare the role of scintigraphy with ^99m^Tc-infliximab in the evaluation of disease with the findings obtained with [^18^F]-FDG PET, but mainly for the selection of patients suitable for treatment with unlabelled anti-TNF*α* mAb. From our data, although on a limited series, it appears that with a qualitative examination, PET confirmed the staging performed by pulmonologists and allowed us to identify locations of extrathoracic disease, like axillaries and abdominopelvic lymph nodes. Inflammatory events in the lung parenchyma of sarcoidosis patients have been confirmed by higher SUV_max⁡_ and SUV_mean_ with respect to normal subjects. The lack of correlation between the CD4^+^/CD8^+^ lymphocytes ratio in BAL and the values of both SUV_max⁡_ and SUV_mean_ in the lung could be explained by the fact that the [^18^F]-FDG is taken up by various cell types involved in the inflammatory sarcoid granuloma, confirming its poor specificity. Alternatively BAL is performed in a single lung segment whereas SUV was calculated over the whole lungs. Scintigraphy with labelled anti-TNF*α* mAb was qualitatively positive in 4 out of 10 patients, showing at 6 h and 24 h a widespread uptake of the radiopharmaceutical in both lungs. The values of* T/B* ratio calculated, on both the 6 h and 24 h images, did not correlate with the values of SUV_max⁡_ and SUV_mean_ calculated on ROI_lung_ of PET.

There are many possible explanations for this different pattern of ^18^F-FDG and ^99m^Tc-infliximab in sarcoidosis patients. One of them is that the two radiopharmaceuticals show different aspects of the same phenomenon: the intense [^18^F]-FDG uptake by the cell populations responsible of alveolitis (macrophages, lymphocytes, etc.) and the presence of TNF*α* revealed by radiolabelled ^99m^Tc-infliximab. Alternatively, there may be individual variability in the production of TNF*α* due to genetic reasons that depend on the stage of the disease. The lack of a clear focal uptake in some patients could also be caused by the long half-life of the anti-TNF*α* mAb that resulted in high background activity from the blood. This phenomenon is critical in highly perfused organs such as the lungs. In this case, the use of isotopes with longer half-life (^89^Zr or ^111^In) could help allowing to acquire images at later time points (e.g., 48 h or 72 h) thus improving* T/B* ratio due to clearance of radiopharmaceutical from the blood. Moreover, it should be kept in mind that, contrarily to what happens in lesions where the targets of a radiopharmaceutical are membrane bound receptors, TNF*α* is also a soluble molecule and a possible washout or low concentration in the lesions could prevent the visualization of distinctive foci. Indeed, on the basis of the review of literature, the most responsive patients to therapy with infliximab appear to be those with extrapulmonary disease, with involvement of skin, nervous system, bone, and ocular disease; it would be interesting to study these patients with ^99m^Tc-infliximab scintigraphy in order to effectively assess the presence of TNF*α* in other extrapulmonary tissues affected by sarcoidosis.

Our choice was to investigate patients with newly diagnosed sarcoidosis that did not undergo prior therapies, based on the need to study “naive” patients in which no previous immunosuppressive therapy was administered. Despite what we expected the* T/B* ratio calculated at 6 h and 24 h did not correlate with the amount of lymphocytes in BAL, indicating that not all immune-mediated phenomena are characterized by high production of TNF*α*.

## 5. Conclusions

Labelled anti-TNF*α* mAb scintigraphy could be a good tool for the selection of patients to be treated with anti-TNF*α* drugs; however, in our study most of the examined patients showed a negative ^99m^Tc-infliximab scintigraphy, underlining a low presence of TNF*α* even if [^18^F]-FDG PET/CT was highly positive.

## Figures and Tables

**Table 1:** Summary of SUV obtained by [^18^F]-FDG PET/CT and *T/B* values (average of right and left lung calculated in anterior and posterior images) in patients with sarcoidosis.

Number/Sex	Stage	SUV_max_	SUV_mean_	*T*/*B* 6 h	*T*/*B* 24 h
1/F	I	1.91	0.54	4.16	2.84
2/F	II	3.47	0.99	4.34	2.75
3/F	II	4.38	2.19	3.52	3.49
4/F	II	2.64	0.79	4.82	3.97
5/M	I-II	3.05	1.02	3.61	3.14
6/F	II	5.28	1.25	4.77	3.24
7/M	I	2.23	0.73	4.14	2.72
8/F	II	12.87	4.55	4.73	3.41
9/F	II	3.01	0.88	3.43	2.49
10/F	II	5.48	0.90	4.73	3.42

Mean ± SD		4.43 ± 3.2	1.38 ± 1.2	4.22 ± 0.55	3.15 ± 0.45
